# A Miniaturised, Fully Integrated NDIR CO_2_ Sensor On-Chip

**DOI:** 10.3390/s21165347

**Published:** 2021-08-08

**Authors:** Xiaoning Jia, Joris Roels, Roel Baets, Gunther Roelkens

**Affiliations:** 1 Photonics Research Group, INTEC, Ghent University-Imec, Technologiepark 126, 9052 Gent, Belgium; Roel.Baets@UGent.be (R.B.); Gunther.Roelkens@UGent.be (G.R.); 2 Center for Nano- and Biophotonics, Ghent University, 9000 Gent, Belgium; 3 Melexis Technologies NV, Transportstraat 1, 3980 Tessenderlo, Belgium; jro@melexis.com

**Keywords:** optical sensor, CO_2_ sensor, NDIR, silicon photonics

## Abstract

In this paper, we present a fully integrated Non-dispersive Infrared (NDIR) CO_2_ sensor implemented on a silicon chip. The sensor is based on an integrating cylinder with access waveguides. A mid-IR LED is used as the optical source, and two mid-IR photodiodes are used as detectors. The fully integrated sensor is formed by wafer bonding of two silicon substrates. The fabricated sensor was evaluated by performing a CO_2_ concentration measurement, showing a limit of detection of ∼750 ppm. The cross-sensitivity of the sensor to water vapor was studied both experimentally and numerically. No notable water interference was observed in the experimental characterizations. Numerical simulations showed that the transmission change induced by water vapor absorption is much smaller than the detection limit of the sensor. A qualitative analysis on the long term stability of the sensor revealed that the long term stability of the sensor is subject to the temperature fluctuations in the laboratory. The use of relatively cheap LED and photodiodes bare chips, together with the wafer-level fabrication process of the sensor provides the potential for a low cost, highly miniaturized NDIR CO_2_ sensor.

## 1. Introduction

CO_2_ gas sensing is of great importance in both academia and industry. A low-cost, miniaturized CO_2_ sensor is desired in many applications such as greenhouse farming, air-quality monitoring, and industrial process control [1,2,3]. For instance, CO_2_ is an important byproduct of the human metabolism. The average CO_2_ concentration in human breath is about 4% and an average person breaths out approximately 1 kg of CO_2_ every day [4]. In an enclosed environment such as schools, workplaces, and residential buildings, human beings are the major source of CO_2_ generation. If poorly ventilated, the exhaled CO_2_ will accumulate and the overall indoor CO_2_ concentration will increase. Although CO_2_ is not considered as a hazardous gas for human beings, intermittent- and long-term exposure to elevated CO_2_ concentrations can lead to a variety of health problems. It is shown that chronic (a few hours) exposure to a CO_2_ concentration of less than 5000 ppm can lead to symptoms such as a decline in cognitive abilities, bone demineralization, and kidney calcification [5]. To mitigate these health risks, sensors are needed to constantly monitor the in-door CO_2_ concentration and to maintain a good indoor air quality through ventilation. Moreover, the outbreak of the Coronavirus disease 2019 (COVID 19) pandemic is expected to create growth opportunities for CO_2_ sensors. In the last few months, there has been rapidly mounting evidence for COVID-19 transmissions via aerosols, which are virus-carrying particles with diameters of several microns that can float in the air for up to a few hours [6,7]. In an indoor environment, the virus-laden aerosols produced by an infected patient can easily accumulate, leading to increased infection risks in multi-occupant spaces. In this regard, ventilation should be considered as part of a hierarchy of risk controls approach. As CO_2_ is co-exhaled with aerosols, indoor CO_2_ concentration has been suggested as a practical indicator of transmission risks of COVID-19 [8,9]. Therefore, measurement of elevated CO_2_ levels by a low-cost CO_2_ sensor is an effective method to identify poor ventilation in an indoor environment and to mitigate the infection risks [10].

The existing gas sensing techniques can be categorized into optical methods and non-optical methods. In terms of CO_2_ sensing, electrochemical sensors and non-dispersive infrared (NDIR) sensors are the main gas sensors dominating the market [11]. Electrochemical sensors measure the gas concentration by measuring a change in electrical properties such as resistance, capacitance, or electric potential induced by the adsorption of the gas [12,13]. They are advantageous because of their easy fabrication, low cost and high sensitivity to a wide range of compounds. However, electrochemical sensors suffer from poor long-term stability and cross-sensitivity to other gases, which make them less attractive in the CO_2_ sensor market [11,14]. On the contrary, NDIR CO_2_ sensors offer superior long-term stability and high gas specificity. The NDIR technique is particularly suited for CO_2_ sensing, as CO_2_ sensing is notoriously difficult with non-optical methods and NDIR opens a new path due to the significant absorption strength of CO_2_ in the mid-IR region. NDIR CO_2_ sensors rely on the very high absorption coefficient of CO_2_ in the mid-infrared wavelength range around 4.26 μm. Due to this high absorption coefficient of CO_2_, the requirement on the interaction length is relaxed and an optical path length of a few centimeters is sufficient to detect small changes in CO_2_ concentration. Due to the aforementioned advantages, NDIR CO_2_ sensors are commercially successful: it is estimated that about 83% of the total advanced CO_2_ sensors are based on the NDIR technique [15]. However, NDIR sensors tend to be bulky as to achieve ppm level detection a long (typically several cm) interaction length is required. The cost of current NDIR sensors is also high as they are typically based on discrete co-assembled optical elements, which limits their application in price- and size-sensitive markets. In recent years, low-cost, highly miniaturized NDIR CO_2_ sensors are receiving increasing attention in both industry and academia, and enormous efforts have been made to achieve a compact and low-cost CO_2_ sensor. To increase the gas–light interaction length while maintaining the compactness of the sensor, the design of the gas cell can be optimized by using either a multi-pass cell [16] or an optical cavity with various shapes [17,18,19]. Apart from these techniques to increase the interaction length, pre-concentrator coatings can be employed to effectively ‘amplify’ the gas concentration in the vicinity of the optical field, thus the required optical path length can be reduced [20]. Yet, the above-mentioned CO_2_ sensors are based on discrete, co-assembled optical elements, leading to an overall high cost of the sensor.

In our previous work [21], we have demonstrated an on-chip NDIR CO_2_ sensor based on an integrating cylinder. Our previously demonstrated integrated cylinder has an equivalent path length of ∼3.5 cm, with a footprint of only 6 × 6 ${\mathrm{mm}}^{2}$. Using an external optical source and photodetectors, a detection limit of ∼100 ppm has been demonstrated for CO_2_ sensing. The response time of the sensor is only 2.8 s, due to the small footprint of the sensor.

The relatively low detection limit, fast response time, and the small footprint of our previously demonstrated CO_2_ sensor give the potential for a low-cost, highly miniaturized and sensitive CO_2_ sensor. Yet, external optical source and detectors were used in our previous work. In this paper, we present a fully integrated on-chip NDIR CO_2_ sensor based on such an integrating cylinder, with the optical source and detector integrated on-chip. The fully integrated CO_2_ sensor consists of an on-chip integrating cylinder as optical cavity, a mid-IR LED emitting at 4.26 μm, and two mid-IR photodiodes integrated on-chip as optical source and detectors. The footprint of the fully integrated sensor is only ∼7 ${\mathrm{mm}}^{2}$. The detection limit of the fully integrated CO_2_ sensor is ∼750 ppm (3σ). The fully integrated sensor shows a diffusion-limited response time of 2.5 s. During the measurement, no notable water interference was observed. Further numerical simulations show that the cross-sensivitity induced by water vapor is below the detection limit of the sensor. A qualitative analysis of the long-term stability of the sensor showed that the response of the fully integrated sensor is temperature dependent, due to the temperature dependence of the LED/photodiodes characteristics.

## 2. Working Principle of NDIR CO_2_ Sensors

**Figure 1:**
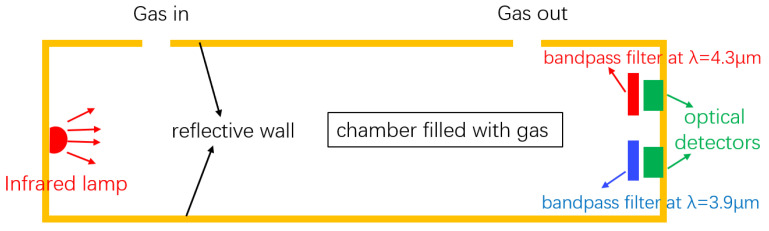
Schematic of a typical NDIR sensor. The sensor consists of an infrared broadband source, a reflecting gas tube, and two optical detectors. The two filters and two detectors form the active channel and reference channel, respectively. Figure adapted from [21].

The structure of a typical NDIR gas sensor is schematically shown in Figure 1. The sensor consists of a broadband infrared source, a reflective gas tube, and two optical detectors. The two detectors act as the two channels (sensing and reference) of the sensor, and the wavelength selection of the two channels is achieved by the optical filters in front of the detectors. For CO_2_ sensing, wavelengths at 4.26 μm and 3.9 μm are typically used at the sensing channel and the reference channel, respectively. The presence of the reference channel is to compensate the light intensity variations due to, e.g., source power fluctuations and waveguide aging; therefore, auto-calibration of the sensor can be achieved. During a CO_2_ sensing measurement, the light at the sensing channel will be absorbed by CO_2_, and the absorption is mathematically described by the Beer–Lambert law:

(1) $$I=I_{0}e^{-\epsilon cL}$$

in which *I* and $I_{0}$ are the light intensity at the output and input, respectively, ϵ is the molar attenuation coefficient, *c* is the CO_2_ concentration, and *L* is the interaction length. From Equation (1), one can calculate the transmission *T* from the source to the detector:

(2) $$T=\frac{I}{I_{o}}=e^{-\epsilon cL}$$

It can be seen that the transmission from the source to the detector can be related to the CO_2_ concentration for a given sensor configuration(fixed *L*). Therefore, it is possible to extract the CO_2_ concentration in the gas sample by measuring the transmission change from the source to the detector.

## 3. Fully Integrated NDIR CO_2_ Sensor

**Figure 2:**
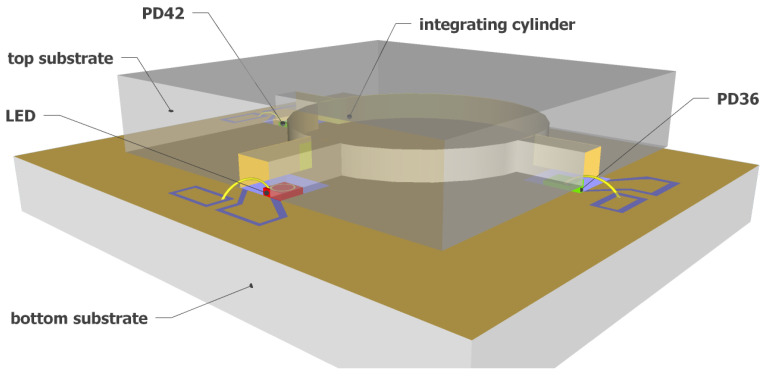
Schematic of the fully integrated on-chip NDIR CO_2_ sensor. The sensor uses an integrating cylinder as sensing cavity, a mid-IR LED as the optical source, and two mid-IR photodiodes (PD42 and PD36) as optical detectors.

Figure 2 shows a 3D schematic of the fully integrated NDIR CO_2_ sensor proposed in this work. A silicon-based integrating cylinder with one input waveguide and two output waveguides is used as the sensing cavity. A surface emitting mid-IR LED is used at the input waveguide of the integrating cylinder as the optical source. The mid-IR LED has a relatively narrow emission spectrum such that the need for optical filters can be eliminated without introducing notable cross-sensitivity by other gases. At the two output waveguides of the integrating cylinder, two mid-IR photodiodes (PD42 and PD36, with “42” and “36” referring to the peak response wavelength of the two photodiodes—4.2 μm and 3.6 μm, respectively) are used as the optical detectors due to their high sensitivity. The two photodiodes are chosen such that the spectral response of sensing photodiode (PD42) has a significant overlap with the CO_2_ absorption band at 4.26 μm, but there is no spectral overlap between the response of the reference photodiode (PD36) and the CO_2_ absorption band. Therefore, the two output waveguides of the integrating cylinder can act as the sensing channel and the reference channel for the fully integrated sensor.

### 3.1. Integrating Cylinder

The integrating cylinder is a direct derivation of an integrating sphere. As in an integrating sphere, the incident light in an integrating cylinder experiences multiple reflections before reaching the detector; therefore, a long effective path length can be obtained on a small sensor footprint. The schematic of the integrating cylinder can also be seen in Figure 2, which is the upper silicon substrate of the fully integrated sensor, consisting of a cylindrical cavity with three access waveguides, one for the input and two for the outputs. The detailed analysis of the integrating cylinder can be found in our previous work [21], in which we have demonstrated a detection limit of ∼100 ppm for CO_2_ sensing using the integrating cylinder and external optical source and detectors. For the fully integrated NDIR CO_2_ sensor proposed in this work, we use an integrating cylinder with access waveguide width *w* = 400 μm to accommodate the dimensions of the LED and photodiodes (as will become clear later). The radius of the cylinder is chosen as *R* = 2.6 mm, such that the sensitivity of the sensor is maximized [21]. Such an integrating cylinder gives an equivalent path length of $L_{eq}$ = 3.4 cm and an input waveguide to output waveguide transmission of −7.2 dB [21].

### 3.2. LED and Photodiode Bare Chips

**Figure 3:**
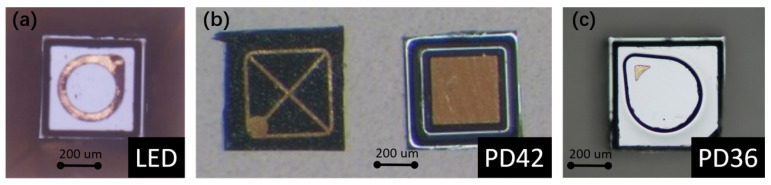
Microscope images of the (**a**) LED, (**b**) PD42 and (**c**) PD36 bare chips. PD42 and PD36 are the photodiodes used in the sensing and reference channels, respectively.

Using an LED as the optical source provides a better power efficiency comparing to a thermal emitter, as its emission spectrum is much narrower than a thermal emitter (broadband, black body radiation). On the detector side, a mid-IR photodiode provides a better responsivity comparing to a thermopile detector. Therefore, the use of an LED-photodiode combination as the source and the detector leads to a lower detection limit of the sensor. Moreover, the need for optical filters can be eliminated due to the relatively narrow emission spectrum of the LED. Figure 3 shows the microscope images of LED and photodiode bare chips used in this work. The dimensions of the LED (LED43 from [22]) and the two photodiodes (PD42 (PD42BS from [23]) for the sensing channel, PD36 (PD36-03 from [22]) for the reference channel) are 400 μm × 400 μm × 250 μm and 500 μm × 500 μm × 200 μm, respectively. The bare chips have a ring/grid/point top contact and a planar bottom contact, through which the chips can be integrated on the silicon substrate with die bonding.

**Figure 4:**
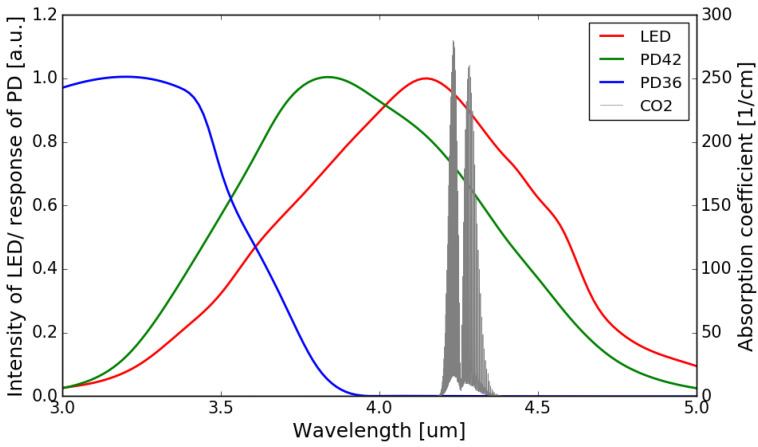
Normalized emission spectrum of the LED (data extrapolated from [22]) and spectral responses of the photodiodes (data extrapolated from [22,23]) with the absorption spectrum of CO_2_ in the 3–5 μm wavelength range (Adapted with permission from ref. [24]. Copyright 2013 Elsevier).

The normalized emission spectrum of the LED and the spectral responses of the sensing photodiode (PD42) and the reference photodiode (PD36) are shown in Figure 4, with the absorption spectrum of CO_2_ at 4.26 μm interposed on top (all at room temperature). It can be seen that the spectral response of the sensing photodiode overlaps with the emission spectrum of the LED and the CO_2_ absorption band, while there is no overlap between the spectral response of the reference photodiode and the CO_2_ absorption band.

## 4. Fabrication

**Figure 5:**
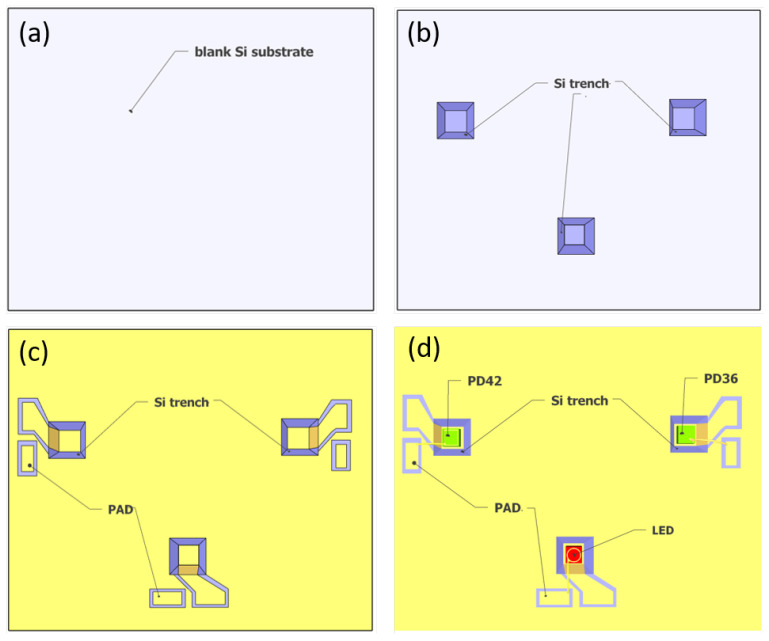
Fabrication process flow of the bottom substrate containing the LED/photodiode chips. (**a**) Blank silicon substrate. (**b**) Three trenches are formed on the substrate via selective etching of silicon by KOH. (**c**) Gold deposition and lift-off. In this step, two contact pads (anode and cathode) are formed for the LED/photodiodes. The deposited gold on the bottom substrate acts as a reflector for the integrating cylinder. (**d**) LED/photodiodes bonding and wire bonding. The LED/photodiode chips are bonded in the trench using die bonding, after which the top contacts of the LED/photodiodes are wire-bonded to the anode pads.

**Figure 6:**
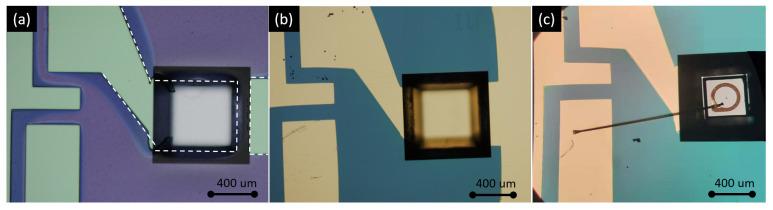
(**a**) Patterned photoresist film (in purple) at the input side, the resist film is hanging over the edge of the trench. (**b**) Contact pads after gold deposition and lift-off. (**c**) Bonded LED chip in the trench, with a wire bond connecting the top contact of the LED and the contact pad on the substrate.

As shown in Figure 2, the sensor is formed by wafer bonding of two silicon substrates. The top substrate of the sensor contains the integrating cylinder, of which the fabrication process has been discussed in our previous work [21]. On the bottom substrate, the LED and photodiodes are integrated. The fabrication process of the bottom substrate is shown in Figure 5. To integrate the LED and photodiodes on-chip, we started with a blank Si substrate (Figure 5a), and the following fabrication steps are performed: (1) trench formation (Figure 5b). Three trenches are formed on the blank silicon substrate by selective etching of silicon with KOH, the etch depth is ∼250 μm, and the widths of the trenches at the bottom surface are designed to be ∼50 μm wider than the widths of the LED/photodiodes; (2) SiO_2_ film deposition. A thin film of SiO_2_ (∼400 nm) is deposited on the substrate using PECVD. The SiO_2_ layer acts as an insulator layer to prevent short circuit between the contact pads, as the silicon substrate is weakly conductive. (3) gold deposition and lift-off. In this step, the gold reflectors on the bottom substrate are formed, as well as the two contact pads (anode and cathode) for the LED/photodiodes. As shown in Figure 5c, both contact pads need to be isolated from the large-area gold reflector on the substrate. Therefore, the inclined sidewalls of the trenches need to be protected from gold deposition. In this context, we used a resist photopolymer film (FX900 series, DuPont) as the protective layer. The large thickness of the resist film (∼20 μm thick) allows it to hang over the trenches. Figure 6a shows the patterned resist film after lithography, and the contact pads after gold deposition and lift-off are shown in Figure 6b. The thickness of the deposited gold is ∼500 nm, with a thin layer (∼50 nm) of Ti for better adhesion; (4) LED/photodiodes bonding and wire bonding (Figure 5d). In this step, the LED/photodiodes bare chips are bonded (with top sides facing up) in the trenches by die bonding. Due to the high roughness of the gold layer on the bottom of the trench and the bottom contacts of the LED/photodiodes (formed by eletroplating of gold), we use a conductive silver adhesive (478SS, from Electron Microscopy Sciences) as bonding agent. After bonding, the adhesive is cured at 160 °C for 30 min. The top contact pads of the LED/photodiodes are then wire-bonded to the anode pads on the silicon substrate. Figure 6c shows a microscope image of the LED bare chip with wire bond. (5) Finally, the sensor is formed by wafer bonding of the top substrate and the bottom substrate. The bonding is performed by gold-to-gold thermocompression bonding (at 160 °C, 1 MPa, for 30 min).

## 5. Experiments

**Figure 7:**
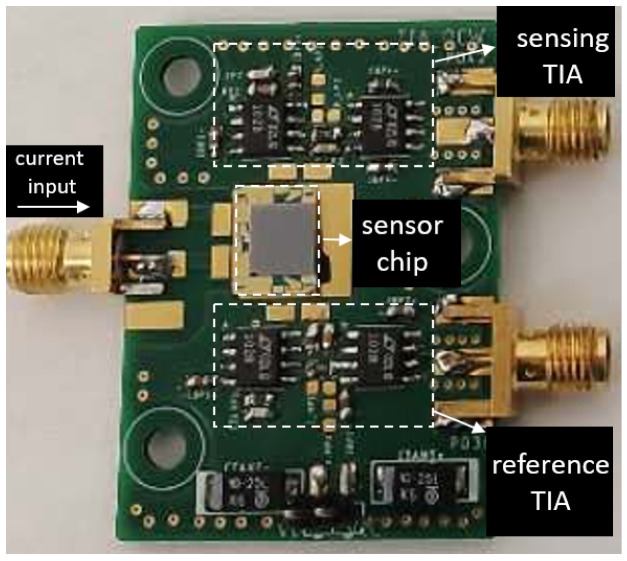
PCB consisting of the TIAs for the sensing photodiode and the reference photodiode. The fabricated sensor chip is mounted on the PCB and the contacts pads of the LED/photodiodes are connected to the board with wire bonds.

To convert the generated photocurrent into voltage, two trans-impedance amplifiers (TIA) are designed for the two photodiodes at the sensing channel and the reference channel. The TIA at the sensing channel has a transimpedence gain of $10^{6}$ Ω and a bandwidth of 2 kHz. For the TIA at the reference channel, we set the gain to be $5\cdot 10^{6}$ Ω, considering the smaller overlap between the spectral response of the reference photodiode and the emission spectrum of the LED (as can be seen in Figure 4). The two TIAs are otherwise identical. The two TIA circuits are then soldered on a printed circuit board (PCB), after which the fabricated sensor chip is mounted on the PCB and the contact pads of the LED/photodiodes are wire-bonded to the contact pads on the PCB. The PCB consisting of two TIAs is shown in Figure 7, with the sensor mounted and wire-bonded on it.

During the measurement, the LED is driven by a pulsed current source (LDP-3811, ILX Lightwave), and the output signals from the TIAs are fed into two lock-in amplifiers (SR380, Stanford Research Systems). The two lock-in amplifiers are triggered by the current source, and they are synchronized and have identical settings during the measurement.

To eliminate the common-mode noise such as source power fluctuations, the signal at the reference arm $I_{R}$ is constantly measured and it is used to normalize the signal at the sensing arm $I_{A}$. The difference in the amplitudes of the two signals is corrected by the factor of $\frac{I_{R0}}{I_{A0}}$, which can be obtained by flushing the sensor with CO_2_-free air at the beginning of the measurement. The normalized signal can thus be written as:

(3) $$S_{norm}=1-\frac{I_{R0}}{I_{A0}}\frac{I_{A}}{I_{R}}$$

In all following measurement results, we use the quantity $S_{norm}$ as a figure of merit to evaluate the sensor performance.

### 5.1. CO_2_ Response

**Figure 8:**
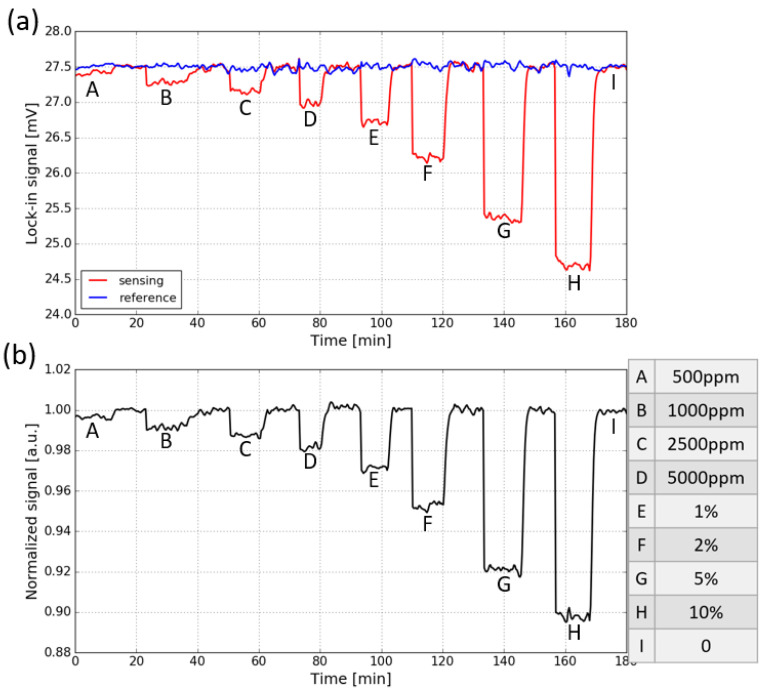
(**a**) Response of both the sensing signal and reference signal to CO_2_ concentration steps, the signals are measured with an integration time of 30 s. (**b**) Normalized transmission, obtained by dividing the sensing signal with the reference signal. The CO_2_ concentration steps are listed on the right side.

The response of the fully integrated sensor to CO_2_ is measured by purging the sensor (with a gas flow rate of ∼10 L/min) with a series of sample gases containing various concentrations of CO_2_ (the sensor is placed in the ambient). In each concentration step, the sensor is first flushed with CO_2_ and then with pure N_2_. The evolution of the two signals when the CO_2_ concentration is varied is shown in Figure 8a. The letters from A to I correspond to different CO_2_ concentrations which are listed on the right side of the figure. The signals are measured with an integration time of 30 s. It can be seen that for the sensing signal, a step response to CO_2_ concentration changes is present, while the reference signal stays relatively stable, which is due to the lack of spectral overlap between the spectral response of the reference photodiode and the CO_2_ absorption spectrum.

**Figure 9:**
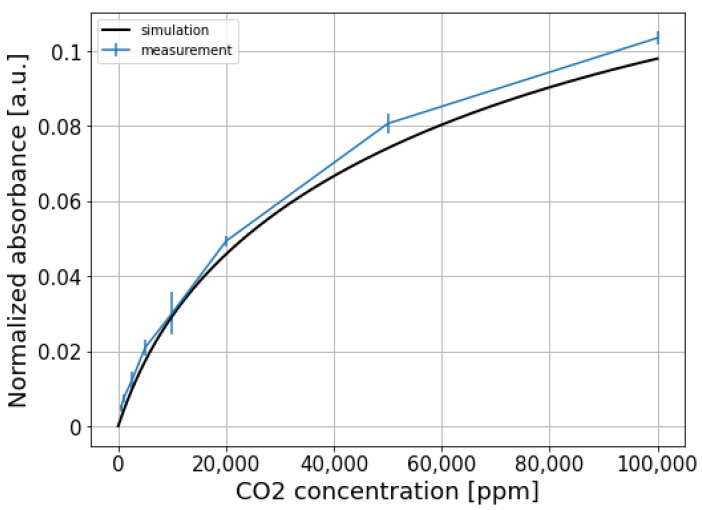
Normalized absorbance of the fully integrated sensor at different CO_2_ concentrations.

To eliminate the common mode noise present on both signals, the sensing signal is normalized w.r.t the reference signal, and the results are shown in Figure 8b. It can be seen that the normalized signal for the fully integrated sensor is still quite noisy. This is because two different photodiodes and TIAs are used at the sensing channel and the reference channel, thus the two channels are not balanced and the common-mode noise cannot be completely eliminated. The normalized absorbance $S_{norm}$ for each CO_2_ concentration step, calculated using Equation (3), is shown in Figure 9. The limit of detection of the fully integrated sensor is about 750 ppm (3σ).

Comparing to our previously demonstrated CO_2_ sensor with external optical source and detectors [21], the detection limit of the fully-integrated sensor is 7.5× worse (750 ppm vs. 100 ppm). The reasons for this deterioration are two-fold: (1) the bandwidth of the LED emission (FWHM = 1000 nm) is larger than the bandwidth of the optical filter (FWHM = 500 nm) used in our previous work, which leads to a larger ’dilution’ of the CO_2_ absorbance over the optical bandwidth; (2) in our previous work, an ultra-stable optical source is used, while there is no stabilization circuit for the LED of the fully integrated sensor. In principle, the power fluctuations of the LED can be eliminated by normalizing the sensing signal w.r.t the reference signal; however, the two arms are not perfectly balanced due to the differences in the sensing arm and the reference arm (photodiodes and TIAs).

The normalized absorbance is also simulated by propagating the emission spectrum of the LED through the absorption spectrum of CO_2_, and the resulting spectrum is weighted by the responsivity function of the sensing photodiode (the emission spectrum of the LED, absorption spectrum of CO_2_, and the responsivity function of the sensing photodiode are all shown in Figure 4). This virtual propagation process can be mathematically described by the following formula:

(4) $$\begin{array}{cc} S_{norm}\left(c\right) & =1-\frac{I_{A}\left(c\right)}{I_{0A}\left(c=0\right)}\\ & =1-\frac{\int_{\lambda_{1}}^{\lambda_{2}}P\left(\lambda \right)\cdot \exp\left[-\alpha \left(\lambda \right)\cdot L\cdot c\right]\cdot R\left(\lambda \right)\;d\lambda }{\int_{\lambda_{1}}^{\lambda_{2}}P\left(\lambda \right)\cdot R\left(\lambda \right)\;d\lambda } \end{array}$$

where $I_{A}$ and $I_{0A}$ are the sensing signals with/without the presence of CO_2_, respectively. The difference of the upper and lower bounds ($\lambda_{1}$ and $\lambda_{2}$) of the integral represents the wavelength range of interest. P(λ) is the emission spectrum of the LED, R(λ) is the responsivity function of the sensing photodiode. The term exp[−α(λ)·L·c] represents the absorption of CO_2_, with α(λ) being the absorption coefficient, and L=3.4 cm and *c* being the equivalent path length and CO_2_ concentration, respectively. It can be seen that the agreement between the measurement and the simulation is reasonably good.

### 5.2. Response Time

In our previous work on an NDIR CO_2_ sensor based on an integrating cylinder (with a radius R=2.0 mm and access waveguide widths d=200 μm), a response time of 2.8 s was demonstrated [21]. For the fully integrated sensor proposed in this work (integrating cylinder with a radius R=2.6 mm and access waveguide widths d= 400 μm), the same experimental setup and procedure is used to measure the response time of the sensor. The sensor is first flushed with a sample gas containing 50% CO_2_, then the gas flow is abruptly shut off, after which the CO_2_ concentration in the sensor reaches ambient level (∼400 ppm) due to gas diffusion. In this measurement we use an integration time of 100 ms to resolve the step of the signal. The response time (T90) of the fully integrated sensor is approximately 2.5 s. However, one should note that a much longer integration time (e.g., 30 s in our CO_2_ sensing measurement) is needed to detect ambient-level CO_2_ concentrations (a few hundreds of ppm). This long integration time also makes the response time of the sensor much longer, although it is usually not a problem for applications such as air quality monitoring.

### 5.3. Water Interference

**Figure 10:**
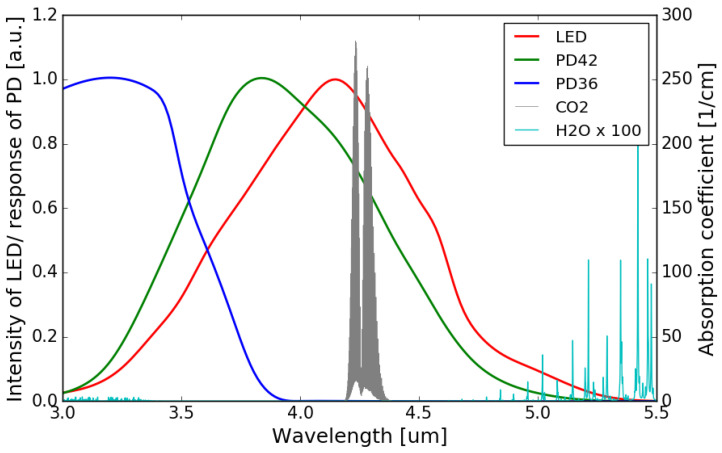
Water vapor and CO_2_ absorption spectra in 3.0–5.5 μm wavelength range (Adapted with permission from ref. [24]. Copyright 2013 Elsevier), together with the emission/responsivity spectra of the LED/photodiodes. The amplitude of the water vapor absorption spectrum is magnified by a factor of 100.

The accuracy of NDIR gas sensors can deteriorate with the presence of water molecules, which largely depends on the relative humidity (*RH*) of the environment. Water vapor has a broad absorption in the infrared wavelength range, and this absorption can be significant at some specific wavelengths [25]. Depending on the spectral overlap between the absorption band of the target gas and the water molecules, NDIR gas sensors exhibit different water interference characteristics. The water interference for an NDIR CO_2_ sensor is typically very small as there is virtually no overlap between the water absorption spectrum and the CO_2_ absorption band at 4.2 μm. Figure 10 shows the absorption spectrum of water vapor and CO_2_, as well as the emission/responsivity spectra of the LED/photodiodes used in this work in the 3.0–5.5 μm wavelength range. It can be seen that the overlaps between the water absorption and the emission spectrum of the LED/spectral responses of the two photodiodes are negligible.

To experimentally evaluate the response of the sensor under different *RH* levels, we introduce a third gas containing pure N_2_ with 100% *RH* (with flow rate of $F_{N2,H2O}$), obtained by flushing pure N_2_ through a bottle with liquid water. Then sample gases with different *RH* levels can be generated by adjusting the flow rates of the three gases:

(5) $$RH=\frac{F_{N_{2},H_{2}O}}{F_{N_{2},H_{2}O}+F_{N_{2}}+F_{CO_{2}}}\times 100\%$$

**Figure 11:**
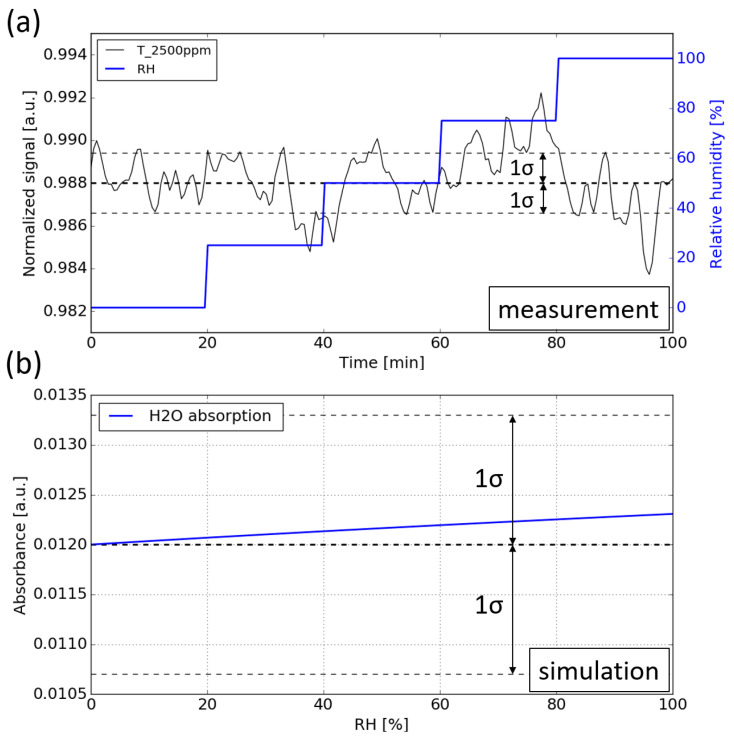
(**a**) Water interference measurement. During the measurement, the RH level is swept from 0 to 99% while the CO_2_ concentration is kept at 2500 ppm. (**b**) Simulation of the water vapor absorption at various RH levels, the absorption of water is added on top of the absorption of 2500 ppm CO_2_.

During the measurement, the CO_2_ concentration is kept constant at 2500 ppm while the *RH* level is swept from 0 to 99%. Figure 11a shows the normalized signal against time. It can be seen that no notable water interference can be observed for the sensor, which means that the cross sensitivity introduced by the water vapor is below the detection limit of the sensor.

To determine the magnitude of the cross-sensitivity introduced by water vapor, numerical simulations are carried out. The simulation procedure is the same as in Section 5.1: propagating the emission spectrum of the LED through the absorption spectra of water vapor (with various *RH* levels), after which the resulting spectrum is ‘sampled’ by the photodiode by calculating its spectral overlap with the spectral response of the sensing photodiode. The absorbance of the water vapor at various RH levels is plotted in Figure 11b. The baseline corresponds to the absorbance of 2500 ppm CO_2_. It can be seen that the absorbance induced by water vapor is within 5 × $10^{-4}$, which is much smaller than the detection limit of the sensor (1σ = 1.2 × $10^{-3}$).

### 5.4. Long Term Stability

**Figure 12:**
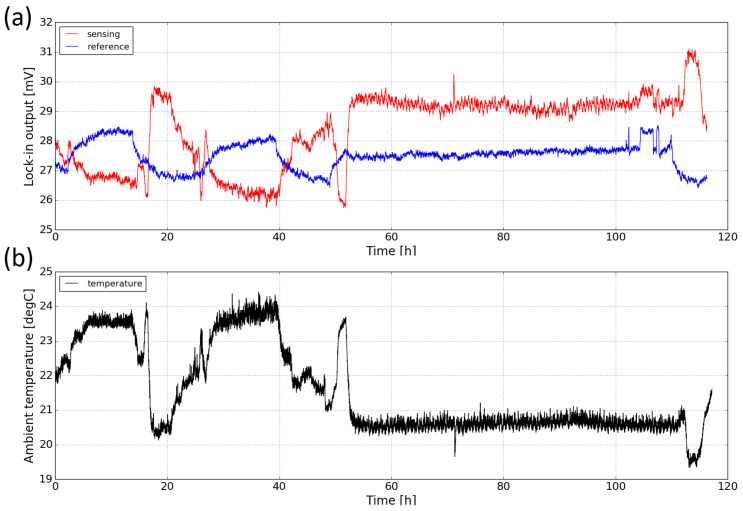
(**a**) Long-term stability measurement of the sensor for a period of ∼five days. (**b**) Ambient temperature profile measured with a thermopile detector. The relatively stable period (from 55 h to 110 h) corresponds to the weekend.

The stability of the sensor (also known as drift) deals with the degree to which the sensor’s characteristics remain constant over time. Drift can be attributed to factors such as temperature fluctuations and component aging. To study the long-term stability of the sensor, we measured the response of the sensor in ambient for a period of ∼ five days. Figure 12a shows both the sensing and the reference signal of the sensor against time. It can be seen that during the measurement, both signals exhibit relatively large scale fluctuations (up to 15%). Moreover, the sensing signal and the reference signal change in opposite directions. It is believed that the changes in both signals are due to the temperature fluctuations in the laboratory. Therefore, the ambient temperature during the course of the measurement is also monitored with a thermopile detector (MLX90632, from Melexis) and the temperature profile is plotted in Figure 12b. One can see that there is a strong correlation between the ambient temperature and the sensing signal. The worse correlation of the reference channel is not yet fully understood.

**Figure 13:**
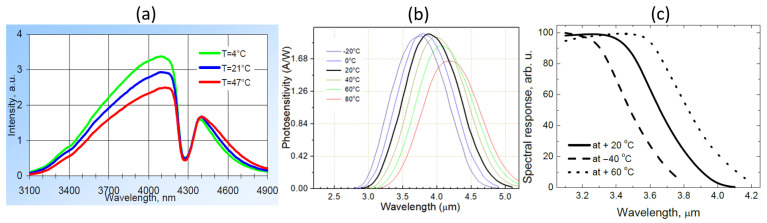
Temperature-dependence of the LED/photodiodes characteristics. (**a**) Emission spectrum of the LED. (**b**) Spectral response of the sensing photodiode. (**c**) Spectral response of the reference photodiode. Figures taken from [22,23,22], respectively.

The opposite correlation between the two signals and the ambient temperature can be explained by the temperature-dependence of the LED and photodiode characteristics as shown in Figure 13. When temperature increases, the light intensity of the LED emission decreases and the spectrum slightly red-shifts. Meanwhile, the response of the sensing photodiode also decreases and the spectrum also red-shifts; these decreases in both LED emission and photodiode response contribute to the decrease of the sensing signal when the temperature is increased. For the reference photodiode, the spectral response also red-shifts and therefore its spectral overlap with the LED emission spectrum increases, leading to an increase of the reference signal. The temperature-dependence of the characteristics of the LED and photodiodes are therefore responsible for the long-term drift of the sensor performance. In future iterations of the sensor, this temperature dependence can be minimized, e.g., by using a thermoelectric cooler (TEC) to control the temperature of the sensor.

## 6. Conclusions and Outlook

In summary, a fully integrated NDIR CO_2_ sensor on-chip is demonstrated in this work. The sensor uses an integrating cylinder as the optical cavity, with which an interaction length of 3.4 cm can be integrated on a chip area of only 6×6 ${\mathrm{mm}}^{2}$. A mid-IR LED and two photodiode bare chips are used as the optical source and detectors. The LED and photodiodes are integrated on the silicon substrate using die bonding. The fully integrated sensor is formed by wafer bonding of two silicon substrates. CO_2_ sensing with the fully integrated sensor was performed, showing a detection limit of ∼750 ppm (3σ). This relatively high detection limit of the sensor largely attributes to the limited coupling efficiencies between the LED and the input waveguide (8%) and between the output waveguide and the photodiodes (30%). We are currently working on improving the coupling efficiencies by optimizing the interface between the waveguide and the LED/photodiodes; therefore, the sensitivity of the sensor can be further improved. Moreover, LED/photodiodes chips with better performance can potentially be used as the source and detectors. For instance, the LED L15893 from Hamamatsu [26] can emit ∼1 mW optical power at 4.3 μm (with an active area of 700 μm by 700 μm). When we scale the active area to 400 μm by 400 μm (the active area of the LED chip used in this work), the optical power is ${\left(0.4/0.7\right)}^{2}\cdot 1$ mW or 330 μW. Therefore, changing the LED alone can lead to an improvement of detection limit by a factor of 33 (330 μW vs. 10 μW). The cross-sensitivity of the sensor to water vapor was studied both experimentally and numerically. No notable water interference was observed in the experimental characterizations. Numerical simulations revealed that the transmission change induced by water vapor absorption is much smaller than the detection limit of the sensor. A qualitative analysis on the long term stability of the sensor was performed. The experimental results showed that the long term stability of the sensor is subject to the temperature fluctuations in the laboratory, which is due to the temperature dependence of the LED/photodiode characteristics. In future iterations of the sensors, the temperature dependence of the sensor can be avoided using, e.g., temperature control.

## Data Availability

Not applicable.
